# Exploring the Diagnostic Potential of Serum Golgi Protein 73 for Hepatic Necroinflammation and Fibrosis in Chronic HCV Infection with Different Stages of Liver Injuries

**DOI:** 10.1155/2019/3862024

**Published:** 2019-09-17

**Authors:** Xiangjun Qian, Sujun Zheng, Leijie Wang, Mingjie Yao, Guiwen Guan, Xiajie Wen, Ling Zhang, Qiang Xu, Xiangmei Chen, Jingmin Zhao, Zhongping Duan, Fengmin Lu

**Affiliations:** ^1^Department of Microbiology & Infectious Disease Center, School of Basic Medical Sciences, Peking University Health Science Center, Beijing 100191, China; ^2^Artificial Liver Center, Beijing Youan Hospital, Capital Medical University, Beijing 100069, China; ^3^Department of Hepatopancreatobiliary Surgery, Henan Cancer Hospital Affiliated to Zhengzhou University, Zhengzhou 450008, China; ^4^Department of Pathology and Hepatology, the 5th Medical Centre, Chinese PLA General Hospital, Beijing 100039, China

## Abstract

**Background and Aim:**

Serum Golgi protein 73 (GP73) is a promising alternative biomarker of chronic liver diseases, but most data are from patients with HBV infection rather than HCV.

**Materials and Methods:**

Two independent cohorts of chronic hepatitis C (CHC) patients from the 5th Medical Centre of the Chinese PLA General Hospital (*n* = 174) and Beijing Youan Hospital (*n* = 120) with different histories of HCV infection were enrolled. The correlations between serum GP73 and other biochemical indices, as well as its correlations with different stages of liver disease progression, were investigated. The receiver operating characteristic (ROC) curve was employed to evaluate the diagnostic potential of serum GP73 for liver necroinflammation and fibrosis, and comparisons of the diagnostic efficiency with traditional indices of hepatic liver injuries were further investigated.

**Results:**

Levels of serum GP73 were found significantly elevated in patients with moderate to severe inflammatory grade (*G* ≥ 2) and/or with advanced fibrotic stages (*F* ≥ 3) in both cohorts (*P* < 0.05, respectively), as compared to those with a normal or mild liver lesion. Further ROC analysis demonstrated that serum GP73 was comparable to serum ALT and AST in diagnosing the liver necroinflammation grade at *G* ≥ 2, but its diagnostic values for advanced fibrosis (*F* ≥ 3) and cirrhosis (*F* = 4) were limited when compared to APRI and FIB-4, and FIB-4 exhibited the best performance. Notably, an obvious elevation of serum GP73 was observed after patients received PEG-IFN and ribavirin treatment.

**Conclusions:**

Serum GP73 is an important biomarker in evaluating and monitoring the disease progression including liver necroinflammation and fibrosis in patients with chronic HCV infection, but the value is limited for diagnosing advanced fibrosis and cirrhosis in comparison with APRI and FIB-4.

## 1. Introduction

About 80~150 million persons are chronically infected with hepatitis C virus (HCV) worldwide [1, 2]. Chronic HCV infection is the major cause of viral hepatitis, which finally progresses into hepatic fibrosis, cirrhosis, and hepatocellular carcinoma, and 350,000 deaths occur each year due to all HCV-related causes [3, 4]. Numerous studies have demonstrated that necroinflammation is a key component and contributor to hepatic wound healing and fibrogenesis [5–7], and the severity of liver fibrosis and cirrhosis is a significant predictor of disease progression and clinical prognosis for patients with chronic hepatic disease. Fortunately, antiviral treatment can reverse the fibrosis or even early cirrhosis [8–11]. To better manage the chronic hepatitis C (CHC) patients, it is critical to evaluate and monitor the grade of inflammation and the stage of liver fibrosis and cirrhosis.

At present, though liver biopsy remains to be the gold standard for grading the activity of inflammation and histological lesions of the disease simultaneously [12, 13], it is not a feasible option because of potential risk of complications, sampling error, and interobserver variability [13–15]. Instead, several noninvasive methods for fibrosis assessment have been proposed as the alternatives to liver biopsy, such as the AST-to-platelet ratio index (APRI), fibrosis index based on four factors (FIB-4), and transient elastography (TE) which are based on blood indices and imaging modalities, respectively [12, 13]. They are relatively inexpensive and commonly accessible in most hospitals but can be affected by many factors like steatosis and cholestasis [16–18].

Golgi protein 73 (GP73) is a 73 kDa transmembrane glycoprotein mainly expressed in biliary epithelial cells but rarely in hepatocytes in normal liver [19]. The expression of GP73 was found significantly enhanced in acute and chronic liver disease [20]. Recently, studies from others and our laboratory have shown that serum GP73 levels were positively correlated with the progression of chronic liver disease, including inflammation and fibrosis/cirrhosis [21–25]. Since previous researches about GP73 were mainly focused on HBV infection-related liver disease, the diagnostic potential of serum GP73 in chronic HCV infection-related disease remains to be investigated.

In the present study, we aimed to explore the correlations between serum GP73 and other biochemical indices among the chronic hepatitis C patients. Then, the diagnostic potential of serum GP73 for liver lesions was evaluated. Its performance was compared with that of alanine aminotransferase (ALT) and aspartate aminotransferase (AST) for identifying hepatic necroinflammation, as well as with that of APRI and FIB-4 models for fibrosis in different cohorts.

## 2. Materials and Methods

### 2.1. Patients

Two independent cohorts (Cohort A and Cohort B) with different histories of HCV infection were included in this retrospective study. Cohort A is composed of 174 inpatients from the 5th Medical Centre of the Chinese PLA General Hospital (PLAGH) between 2012 and 2017, including 96 patients with precirrhotic CHC, 35 cases with compensated liver cirrhosis (CLC), and 43 cases with decompensated liver cirrhosis (DLC). The demographics, biopsy results, and laboratory data including levels of serum GP73 of these patients were collected (Table 1). Cohort B from Beijing Youan Hospital had been detailed in prior research [26]. In brief, Cohort B including 120 patients, which belong to the Chinese Han ethnicity from rural villages in Dingxi City, suffered from HCV infection through regular plasma donations with repeated blood retransfusions between 1992 and 1995. All of them received a comprehensive examination including drawing cubital vein blood under the fast and accepting liver biopsy from July 2010 to June 2011; then, serological indicators were tested and histological results were evaluated (Table 2).

All participants had accepted liver biopsy to assess the progression of liver disease, and the biopsies of 10 patients with CLC and 38 patients with DLC from Cohort A were achieved when they were undergoing splenectomy because of low thrombocytopenia, esophageal varices, or bleeding resulting from liver cirrhosis. All of them were treatment-naïve patients. The diagnosis of CHC was in accordance with established criteria [27, 28], and the DLC was defined as cirrhosis with ascites, esophagogastric variceal hemorrhage, encephalopathy, and other serious complications in the past and now. Exclusion criteria are (1) acute hepatitis C, (2) coinfection with hepatitis B virus or other hepatitis viruses, and (3) evidence of hepatocellular carcinoma, nonalcoholic fatty liver disease, and autoimmune liver disease and patients with metabolic or genetic disease and alcohol- or drug-induced liver injury. In addition, another 87 healthy individuals were included as the normal control. They were negative for HBV, HCV, and HIV, without other acute or chronic disease, diabetes, hyperlipidemia, and hypertension and with BMI ≤ 28. And their levels of serum GP73 were quantified.

Informed consent was obtained from all participants above. All procedures performed in this study involving human participants were in accordance with the ethical standards of the institutional and/or national research committee and with the 1964 Helsinki declaration and its later amendments or comparable ethical standards.

### 2.2. Liver Histology

The liver specimens were fixed in formalin and embedded in paraffin and then stained with hematoxylin-eosin (HE) and Masson stains. Pathological analyses of the liver biopsies from all the two cohort patients were performed. The Scheuer scoring system (G0-4) was used for the evaluation of histological necroinflammatory activity. To evaluate the hepatic fibrosis stage, the METAVIR scoring system was used which was assessed on a five-point scale: F0, no fibrosis; F1, portal fibrosis without septa; F2, few septa; F3, numerous septa without cirrhosis; and F4, cirrhosis. ≥F2 was defined as significant fibrosis, and ≥F3 was defined as advanced fibrosis. All liver specimens were graded and staged by the same experienced liver pathologist.

Notably, because of having serious liver cirrhosis in 26 cases with CLC and all DLC cases from the 5th Medical Centre of PLAGH (Table 1), they were recruited in another pathological evaluation system of liver cirrhosis without stratified grading of hepatic necroinflammatory activity [29] and were diagnosed with active liver cirrhosis. In this evaluation system [29], liver cirrhosis was divided into active cirrhosis and inactive cirrhosis, in which the former was cirrhosis with obvious inflammation, including inflammation in the fibrous septum, debris necrosis around the pseudolobule, and inflammatory lesions in regenerative nodules.

### 2.3. Measurement of Serum GP73 Levels

Serum GP73 levels were quantified by using commercially available enzyme-linked immunosorbent assay (ELISA) kits (Hotgen Biotech, Beijing, China), according to the manufacturer's protocol.

### 2.4. Treatment and Follow-Up

In Cohort A, there were 24 patients who got a standardized antiviral therapy by PEGylated interferons (PEG-IFN) plus ribavirin and were followed up in the 5th Medical Centre of PLAGH. Serum GP73 levels were monitored from 6 months to 12 months after the treatment; most of them got complete early or partial virological response.

### 2.5. Statistical Analyses

Statistical analyses were performed by SPSS 22.0 software (International Business Machines Corporation, New York, USA) and GraphPad Prism version 5.0 (GraphPad Software Inc., La Jolla, California). Continuous variables were expressed as mean ± standard deviation (SD) or median and interquartile range (IQR). The differences of groups were analyzed by *t*-test, ANOVA, or Kruskal-Wallis rank sum test according to the data's distribution. In addition, the chi-square test was applied to compare the rates of the classification data. Pearson and Spearman's rank correlation coefficient tests were used to describe the association between two variables. The diagnostic effectiveness of variables was assessed by the area under the receiver operating characteristic (AUROC) curve with 95% confidence interval (CI), sensitivity, specificity, positive predictive value (PPV), and negative predictive value (NPV), and the differences were tested by Hanley and McNeil. All tests of significance were two-tailed, and *P* < 0.05 was considered statistically significant.

## 3. Results

### 3.1. Clinical Characteristics of Patients

The clinical characteristics of the Cohort A patients are shown in Table 1. There was no significant difference in gender, body mass index (BMI), HCV RNA, and ALT among the three subgroups (*P* > 0.05). Table 2 shows the baseline characteristics of the Cohort B patients and simultaneous comparison with the Cohort A patients in which the DLC patients were excluded for further study. Significant differences (*P* < 0.05) were observed in BMI, HCV RNA, total bilirubin (TBIL), AST, gamma glutamyltransferase (GGT), albumin (ALB), globulin (GLB), red blood cell (RBC), platelet (PLT), prothrombin time (PT), necroinflammatory activity grade, and fibrosis stage between two groups which had absolutely different background of epidemiology. Besides, the 87 healthy individuals enrolled in this study included 14 males and 73 females; their mean age, BMI, and GP73 were 47.08 ± 7.89 years, 23.31 ± 2.36, and 31.37 ± 14.46 ng/mL, respectively, and the median of ALT was 11.0 U/L.

### 3.2. Serum GP73 Levels Are Gradually Increased along with the Liver Disease Progression of Chronic Hepatitis C Patients

**Figure 1:**
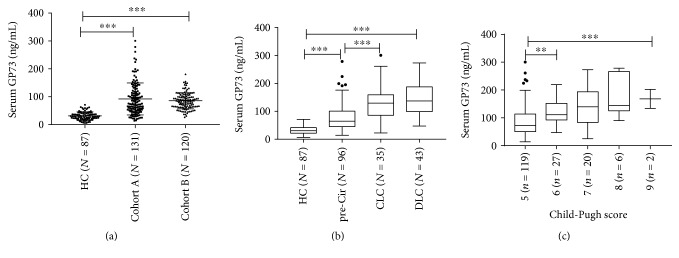
Serum Golgi protein 73 (GP73) levels were gradually increased along with liver disease progression in patients with chronic HCV infection. (a) Comparison of serum GP73 levels in the healthy control (HC), Cohort A, and Cohort B. (b) Comparison of serum GP73 levels in the healthy control (HC), precirrhotic chronic hepatitis C (pre-Cir CHC), compensated liver cirrhosis (CLC), and decompensated liver cirrhosis (DLC) patients in Cohort A. (c) Comparison of serum GP73 levels in different scores of Child-Pugh. ^∗^*P* < 0.05, ^∗∗^*P* < 0.01, and ^∗∗∗^*P* < 0.001.

The serum levels of GP73 (*M* ± SD) were 92.22 ± 57.26 ng/mL in Cohort A without DLC patients and 85.80 ± 29.09 ng/mL in Cohort B, and all were significantly higher than 31.37 ± 14.46 ng/mL in the healthy controls (*P* < 0.001, Figure 1(a)). In Cohort A, though there were no statistically significant differences between the compensatory LC and decompensated LC groups (*P* = 0.615), the serum GP73 levels were significantly higher in the CLC subgroup (131.51 ± 61.97 ng/mL) and DLC subgroup (140.22 ± 55.02 ng/mL) than in the precirrhotic CHC subgroup (77.90 ± 48.34 ng/mL) (*P* < 0.001, Figure 1(b)). Furthermore, levels of serum GP73 were increasingly elevated with the worsening of the Child-Pugh classification scores, and the differences between these subgroups with different scores were statistically significant (*P* < 0.001, Figure 1(c)).

### 3.3. Serum GP73 Levels Are Correlated with the Clinical Indices of Liver Injury, Fibrosis, and Function

The correlations between the levels of serum GP73 and the biochemical indices and pathological indices reflecting the liver injury, fibrosis, and function were then analyzed. As shown in Table 3, in Cohort A, the levels of serum GP73 showed significant correlations with those inflammatory injury indexes, such as AST, GGT, ALB, and prealbumin (PA), and strong correlations with some others like total bile acid (TBA), PLT, and PT. Meanwhile, serum GP73 also exhibited strong positive correlations with histological lesions of the liver, such as fibrotic scores and necroinflammatory activity grade. Concordantly, significant positive correlations with APRI and FIB-4 were observed. The similar results were also obtained in Cohort B, although they appeared as a relatively weaker correlation than Cohort A (Table 3).

### 3.4. A Stepwise and Significant Increase in Serum GP73 Levels Was Observed along with Necroinflammation and Fibrosis Disease Progression

**Figure 2:**
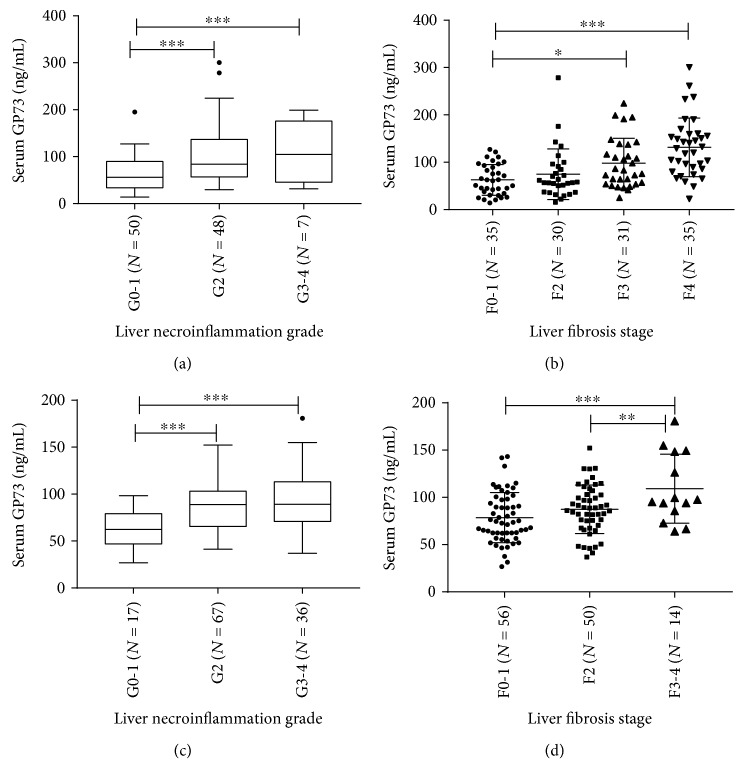
Serum Golgi protein 73 (GP73) levels were elevated obviously along with liver necroinflammation grade and fibrosis stage. (a, c) The correlation between serum GP73 levels and liver necroinflammation grade in Cohort A and Cohort B, respectively. (b, d) The correlation between serum GP73 levels and liver fibrosis stage in Cohort A and Cohort B, respectively. ^∗^*P* < 0.05, ^∗∗^*P* < 0.01, and ^∗∗∗^*P* < 0.001.

In order to further investigate the potential capability of serum GP73 in predicting liver necroinflammation and fibrosis of chronic hepatitis C patients, the levels of serum GP73 in patients with different necroinflammatory grades and fibrotic scores were analyzed in patients without DLC. The serum GP73 levels (*M* ± SD) of Cohort A were 62.42 ± 35.33 ng/mL (G0-1), 101.47 ± 60.39 ng/mL (G2), and 104.28 ± 63.74 ng/mL (G3-4) in each grade of necroinflammation and were 62.80 ± 32.51 ng/mL (F0-1), 74.72 ± 53.42 ng/mL (F2), 98.02 ± 52.42 ng/mL (F3), and 131.51 ± 61.97 ng/mL (F4) in each stage of fibrosis, which indicates that serum GP73 was correlated tightly with the grade of liver necroinflammation (*n* = 105) and with the stage of fibrosis (*n* = 131) (*P* < 0.001, respectively) (Figures 2(a) and 2(b)). Correspondently in Cohort B, the values (*M* ± SD) in each inflammatory grade were 61.26 ± 20.91 ng/mL (G0-1), 87.51 ± 25.30 ng/mL (G2), and 94.22 ± 32.42 ng/mL (G3-4) (*P* < 0.001, Figure 2(c)) and in each fibrotic stage were 78.56 ± 26.51 ng/mL (F0-1), 87.36 ± 25.68 ng/mL (F2), and 109.25 ± 36.46 ng/mL (F3-4), respectively (*P* = 0.001, Figure 2(d)). The differences among different grades were always statistically significant at *G* ≥ 2 or not, as well as different stages at *F* ≥ 3 or not (*P* < 0.05), in the two cohorts.

### 3.5. The Diagnostic Value of Serum GP73 for Moderate Liver Inflammation (*G* ≥ 2), Advanced Fibrosis, and Cirrhosis in Chronic Hepatitis C Patients

Since the increases of serum GP73 levels were always statistically significant in patients with inflammatory grade *G* ≥ 2, as well as with fibrotic scores at *F* ≥ 3 (*P* < 0.05), in the two cohorts, then the AUC analysis was conducted to explore the relevant diagnostic potential of this serum marker. As expected, serum GP73 exhibited potential ability to identify patients with liver necroinflammatory grade (*G* ≥ 2) and/or with advanced fibrosis (*F* ≥ 3) and cirrhosis (*F* = 4). However, such diagnostic efficiency in chronic hepatitis C patients was not superior to the traditional biomarkers and models, such as ALT, AST, APRI, and FIB-4. In Cohort A, the AUROC values for serum GP73, ALT, and AST to identify liver necroinflammatory grade 2 or beyond (*G* ≥ 2) were 0.717, 0.685, and 0.782 (Table 4), respectively, without significant differences (*P* > 0.05). Meanwhile, the AUROC values for serum GP73, APRI, and FIB-4 were 0.761, 0.796, and 0.848, respectively, when predicting advanced fibrosis (*F* ≥ 3), and were 0.779, 0.836, and 0.904, respectively, when predicting cirrhosis (*F* = 4) (Table 5), and also, there were no statistic differences between them. Similar results were obtained in Cohort B; the AUROC values for serum GP73, ALT, and AST to diagnose moderate and severe liver necroinflammatory activities (*G* ≥ 2) were 0.794, 0.777, and 0.769, respectively (Table 4); and the AUROC values for serum GP73, APRI, and FIB-4 were 0.709, 0.839, and 0.829, respectively, when used to diagnose advanced fibrosis (*F* ≥ 3) (Table 5), and their diagnostic efficiency was similar to each other.

### 3.6. Serum GP73 Levels Were Obviously Elevated in Patients after Receiving PEG-IFN and Ribavirin Treatment

**Figure 3:**
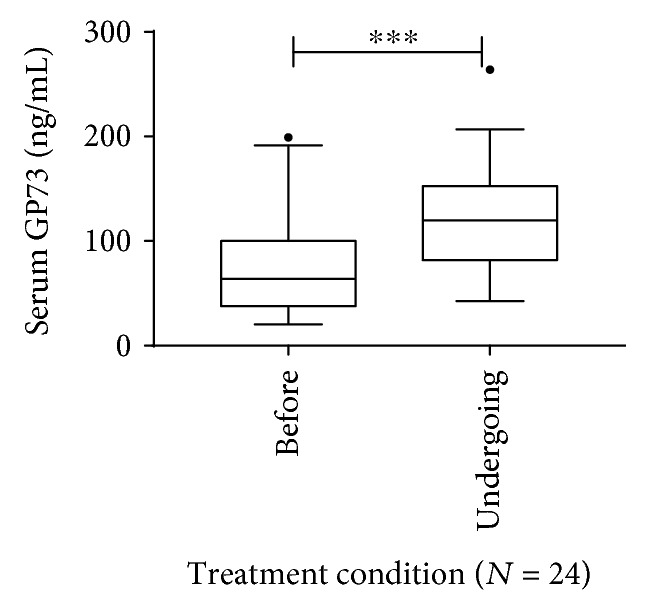
Comparison of serum Golgi protein 73 (GP73) levels in patients with chronic HCV infection before and undergoing (6 months to 12 months after initiating the therapy) treatment with PEGylated interferons (PEG-IFN) and ribavirin. ^∗^*P* < 0.05, ^∗∗^*P* < 0.01, and ^∗∗∗^*P* < 0.001.

Since the level of serum GP73 was closely correlated with liver inflammation and interferon is an immune modulator, therefore, the influence of PEG-IFN and ribavirin treatment on serum GP73 was investigated. For these, indeed, after accepting PEG-IFN and ribavirin treatment, the serum GP73 levels increased remarkably among the 24 patients who got complete early or partial virological response in Cohort A, increasing from 80.21 ± 52.82 ng/mL at baseline to 122.95 ± 50.55 ng/mL after receiving a median of 6 months of treatment, and the changes were statistically significant (*P* < 0.001, Figure 3). Noticeably then in four of them who achieved sustained virological response (SVR, defined as the quantitative HCV RNA undetectable), the levels of serum GP73 decreased to 59.31 ± 10.10 ng/mL at median 6 months after stopping treatment.

## 4. Discussion

Elevated expression of the GP73 protein was reported early in patients with giant-cell hepatitis and adenovirus infection [30, 31], and recent studies further revealed that hepatocyte expression of GP73 was dramatically upregulated in acute and chronic diseased livers, regardless of the etiology [20, 21, 31, 32]. Furthermore, serum GP73 levels were found significantly increased in hepatic necroinflammation, fibrosis, and cirrhosis [21–25, 33–36] and deemed to be an effective and reliable serological surrogate for the diagnosis of advanced fibrosis and cirrhosis and for monitoring the progression of the liver injury and diseases [21–23, 25]. However, the above results were mainly coming from the studies on HBV-related liver diseases, and related data of HCV are absent. The current study was designed to study the role of serum GP73 levels in HCV-related liver disease at different stages except for HCC. Our primary result showed that serum GP73 levels were dramatically elevated in chronic HCV patients compared with healthy controls (Figure 1(a)) and increased quantitatively in a stepwise manner in patients from precirrhotic CHC to CLC and to DLC (Figure 1(b)), as well as the increase in Child-Pugh classification scores reflecting the status of liver injury and residual function (Figure 1(c)). This result is consistent with the results of studies in HBV-related liver disease [24, 37, 38].

As known, serum ALT, AST, GGT, and TBA are commonly used indicators of liver injury; PLT levels are associated with liver fibrosis and cirrhosis [39]; while TBIL, ALB, and PT are the members of the Child-Pugh classification reflecting liver reserve function, these and PA are connected tightly with liver synthesis. Our results revealed that serum GP73 levels were negatively correlated with ALB, PA, and PLT levels, but positively correlated with ALT, AST, GGT, TBA, and PT levels and necroinflammation activity grade, fibrosis stage, and scores of APRI and FIB-4 (Table 3). All these indicated that serum GP73 levels were tightly associated with liver necroinflammation, fibrosis, and function. Moreover, the relationship between the variation of GP73 levels and lesions of HCV-related liver disease was investigated. Levels of serum GP73 rose in parallel with the severity of hepatitis and fibrosis, from nonexistent or mild to moderate and severe necroinflammation and fibrosis, even cirrhosis. These findings are similar to the recent reports demonstrating elevation of GP73 levels in HBV infection-related liver disease progression [22–24, 31, 37, 38].

Though some studies have proven that serum GP73 was a valuable candidate for diagnosing advanced fibrosis and cirrhosis [21, 23, 38, 40] and may even have a higher diagnostic value compared to the traditional noninvasive indices such as APRI, FIB-4, and liver stiffness measurement (LSM) [21, 40], some other studies prefer it to be a new effective biomarker for diagnosing liver necroinflammation [22, 24, 37]. Noticeably, all these results are mainly from patients with HBV-related liver disease. The results of the current study demonstrated that serum GP73 levels were comparable to serum ALT and AST in assessing the moderate to severe liver necroinflammation (*G* ≥ 2) of chronic hepatitis C patients, but its power in diagnosing advanced fibrosis (*F* ≥ 3) and cirrhosis (*F* = 4) showed no advantage, as compared to APRI and especially FIB-4. These results differ from those of certain studies in the literature whose study groups had HBV-related infection. It was reported that GP73 is upregulated by HCV infection and mainly enhances HCV secretion through upregulating apolipoprotein E (APOE) [41]. If the possibility of existing differences in selected cases was ruled out, we speculate that it is the different regulatory mechanisms underlying GP73 expression and secretion in patients with HCV infection that result in abnormally elevated serum GP73 and then lead to the damage of diagnostic efficacy in predicting liver inflammation and fibrosis.

At the same time, our results also revealed that serum GP73 levels were obviously elevated when treatment-naïve patients accepted persistent PEG-IFN and ribavirin treatment and would then decrease to a certain level, which still remained higher than those in healthy individuals, after they achieved SVR and stopped treatment. The result suggests that serum GP73 could not truly reflect the situation of liver injury when used in clinical practice for patients with HCV infection treated by PEG-IFN and ribavirin and clinicians should be more cautious.

Limitations of our study are related to the following: (A) It is a retrospective cohort for data from the 5th Medical Centre of PLAGH which might be influenced by unmeasured potential biases. (B) This study included only two centers and the number of cases was limited. (C) There was an obvious difference of the cutoff values of GP73 for diagnosing severe necroinflammatory activities (*G* ≥ 2) between Cohort A and Cohort B, which were 103.5 ng/mL and 64.43 ng/mL, respectively; such difference was also observed in diagnosing advanced fibrosis (*F* ≥ 3), which were 63.89 ng/mL and 93.74 ng/mL, respectively. As shown in Figure 2, these differences might be caused by differences between the two cohorts on numbers of patients at different disease progression stages of necroinflammation and fibrosis. The two cohorts had different situations of natural history of HCV infection, and the different cutoff values would reflect the real states of the two cohorts. It suggested that multicentered prospective studies with a lager cohort are necessary in the future to evaluate the diagnostic potential of serum GP73.

## 5. Conclusion

Serum GP73 is an important indicator in evaluating and monitoring the disease progression including liver necroinflammation and fibrosis in patients with chronic HCV infection. Serum GP73 has a certain value in diagnosing liver necroinflammation (*G* ≥ 2). However, it is limited for serum GP73 to diagnose advanced fibrosis (*F* ≥ 3) and cirrhosis (*F* = 4) in comparison with APRI and FIB-4.

## Figures and Tables

**Table 1:** Demographic and clinical characteristics of different states of chronic HCV infection in Cohort A.

Parameters	Cohort A			*P* value
	Precirrhotic CHC (*n* = 96)	Compensated LC (*n* = 35)	Decompensated LC (*n* = 43)	
Age (y, *M* ± SD)	47.01 ± 12.31	55.51 ± 8.32	53.09 ± 9.37	<0.001
Gender (M/F)	45/51	11/24	17/26	0.268
BMI (kg/m^2^, *M* ± SD)	23.94 ± 3.64	25.07 ± 2.55	23.60 ± 3.30	0.146
HCV genotype (1b/2a/unknown)	45/37/14	17/9/9	11/11/21	0.001
HCV RNA (+/−)	88/8	33/2	37/6	0.417
TBIL (*μ*mol/L)	12.4 (8.9, 16.2)	15.5 (10.0, 24.6)	18.2 (15.2, 22.3)	<0.001
ALT (U/L)	45.5 (25.0, 84.8)	42.0 (28.0, 73.0)	38.0 (22.0, 61.0)	0.347
AST (U/L)	37.5 (25.0, 57.8)	66.0 (46.0, 90.0)	40.0 (34.0, 75.0)	0.001
GGT (U/L)	26.0 (16.3, 52.5)	57.0 (34.0, 100)	32.0 (20.0, 48.0)	<0.001
ALB (g/L, *M* ± SD)	40.18 ± 4.00	36.91 ± 4.15	33.81 ± 3.99	<0.001
GLB (g/L)	30.0 (26.0, 32.3)	35.0 (30.0, 39.0)	31.0 (25.0, 36.0)	0.001
PA (mg/L, *M* ± SD)	179.64 ± 44.19	130.94 ± 47.20	99.78 ± 30.94	<0.001
RBC (10^12^/L, *M* ± SD)	4.49 ± 0.47	4.21 ± 0.48	3.51 ± 0.76	<0.001
PLT (10^9^/L, *M* ± SD)	173.61 ± 59.19	92.51 ± 47.50	57.72 ± 37.69	<0.001
PT (s, *M* ± SD)	11.0 (10.5, 11.5)	1.24 (11.6, 13.1)	13.3 (12.4, 14.1)	<0.001
GP73 (ng/mL, *M* ± SD)	77.90 ± 48.34	131.51 ± 61.97	140.22 ± 55.02	<0.001
Necroinflammation activity grade (G: 0-1/2/3/4)	50/40/6/0	-/8/1/-	—	—
Fibrosis stage (F: 0-1/2/3/4)	35/30/31/0	0/0/0/35	0/0/0/43	—

Continuous variables were expressed as mean ± standard deviation (SD) or median and interquartile range (IQR). BMI: body mass index; HCV: hepatitis C virus; TBIL: total bilirubin; ALT: alanine aminotransferase; AST: aspartate aminotransferase; GGT: gamma glutamyltransferase; ALB: albumin; GLB: globulin; PA: prealbumin; RBC: red blood cell; PLT: platelet; PT: prothrombin time; GP73: Golgi protein 73.

**Table 2:** Demographic and clinical characteristics of the two cohorts.

Parameters	Cohort A (*n* = 131)	Cohort B (*n* = 120)	*P* value
Age (y, *M* ± SD)	49.28 ± 11.96	51.33 ± 7.33	0.438
Gender (M/F)	56/75	57/63	0.450
BMI (kg/m^2^, *M* ± SD)	24.25 ± 3.40	22.34 ± 2.73	<0.001
HCV genotype (1b/2a/unknown)	62/46/23	45/52/23	0.274
HCV RNA (+/−)	121/10	100/20	0.028
TBIL (*μ*mol/L)	12.9 (9.5, 18.4)	15.3 (11.2, 19.1)	0.014
ALT (U/L)	43.0 (26.0, 81.0)	37.6 (29.4, 65.9)	0.684
AST (U/L)	48.0 (29.0, 69.0)	35.4 (28.1, 47.7)	0.024
GGT (U/L)	34.0 (18.0, 66.0)	15.4 (12.3, 25.1)	<0.001
ALB (g/L, *M* ± SD)	39.30 ± 4.28	43.21 ± 2.36	<0.001
GLB (g/L)	31.0 (27.0, 35.0)	27.2 (25.0, 30.4)	<0.001
PA (mg/L, *M* ± SD)	166.63 ± 49.78	171.41 ± 36.96	0.392
RBC (10^12^/L, *M* ± SD)	4.41 ± 0.49	4.76 ± 0.67	<0.001
PLT (10^9^/L, *M* ± SD)	151.95 ± 66.69	171.36 ± 53.20	0.011
PT (s)	11.3 (10.6, 12.1)	11.5 (11.0, 11.9)	0.003
GP73 (ng/mL, *M* ± SD)	92.22 ± 57.26	85.80 ± 29.09	0.573
Necroinflammation activity grade (G: 0-1/2/3/4)	50/48/7/-	17/67/34/2	<0.001
Fibrosis stage (F: 0-1/2/3/4)	35/30/31/35	56/50/12/2	<0.001

Continuous variables were expressed as mean ± standard deviation (SD) or median and interquartile range (IQR). BMI: body mass index; HCV: hepatitis C virus; TBIL: total bilirubin; ALT: alanine aminotransferase; AST: aspartate aminotransferase; GGT: gamma glutamyltransferase; ALB: albumin; GLB: globulin; PA: prealbumin; RBC: red blood cell; PLT: platelet; PT: prothrombin time; GP73: Golgi protein 73.

**Table 3:** Correlation between serum GP73 level and clinical characteristics (rho).

Parameters	Cohort A (*n* = 131)		Cohort B (*n* = 120)	
	Rho	*P* value	Rho	*P* value
Parameters for liver injury				
ALT	0.262	0.003	0.258	0.004
AST	0.526	<0.001	0.278	0.002
GGT	0.557	<0.001	0.186	0.041
TBA	0.378	<0.001	0.180	0.049
Necroinflammation activity grade (*G*)	0.364	<0.001	0.297	0.001
Parameters for liver fibrosis				
PLT	-0.373	<0.001	-0.392	<0.001
Fibrosis stage (*F*)	0.484	<0.001	0.269	0.003
APRI	0.584	<0.001	0.345	<0.001
FIB-4	0.580	<0.001	0.380	<0.001
Parameters for liver function				
TBIL	0.136	0.122	0.009	0.924
ALB	-0.431	<0.001	-0.389	<0.001
PT	0.374	<0.001	0.228	0.012
PA	-0.537	<0.001	-0.464	<0.001

GP73: Golgi protein 73; ALT: alanine aminotransferase; AST: aspartate aminotransferase; GGT: gamma glutamyltransferase; TBA: total bile acid; PLT: platelet; APRI: AST-to-platelet ratio index; FIB-4: fibrosis index based on four factors; TBIL: total bilirubin; ALB: albumin; PT: prothrombin time; PA: prealbumin.

**Table 4:** Diagnostic values of GP73, ALT, and AST for liver inflammatory activity (*G* ≥ 2).

Parameters	AUC	95% CI	Cutoff value	Sensitivity (%)	Specificity (%)	PPV (%)	NPV (%)	*P* value
*G* ≥ 2 (Cohort A)								
GP73	0.717	(0.620, 0.800)	103.5	43.64	92.00	85.7	59.7	<0.0001
ALT	0.685	(0.587, 0.772)	40	69.09	64.00	67.9	65.3	0.0005
AST	0.782	(0.691, 0.857)	40	69.09	74.00	74.5	68.5	<0.0001
*G* ≥ 2 (Cohort B)								
GP73	0.794	(0.710, 0.862)	64.43	82.52	64.71	93.4	37.9	<0.0001
ALT	0.777	(0.692, 0.848)	40	58.25	82.35	95.2	24.6	<0.0001
AST	0.769	(0.683, 0.841)	40	44.66	88.24	95.8	20.8	<0.0001

GP73: Golgi protein 73; ALT: alanine aminotransferase; AST: aspartate aminotransferase; AUC: area under the curve; CI: confidence interval; PPV: positive predictive value; NPV: negative predictive value.

**Table 5:** Diagnostic values of GP73, APRI, and FIB-4 for advanced fibrosis (*F* ≥ 3) and cirrhosis (*F* = 4).

Parameters	AUC	95% CI	Cutoff value	Sensitivity (%)	Specificity (%)	PPV (%)	NPV (%)	*P* value
*F* ≥ 3 (Cohort A)								
GP73	0.761	(0.678, 0.831)	63.89	81.82	58.46	66.7	76.0	<0.0001
APRI	0.796	(0.717, 0.861)	1.5	45.45	84.62	75.0	60.4	<0.0001
FIB-4	0.848	(0.775, 0.905)	3.25	59.09	87.69	83.0	67.9	<0.0001
*F* = 4 (Cohort A)								
GP73	0.779	(0.698, 0.847)	64.96	91.43	52.08	41.0	94.3	<0.0001
APRI	0.836	(0.761, 0.895)	2.0	51.43	90.62	66.7	83.7	<0.0001
FIB-4	0.904	(0.840, 0.948)	3.25	85.71	84.37	66.7	94.2	<0.0001
*F* ≥ 3 (Cohort B)								
GP73	0.709	(0.619, 0.788)	93.74	71.43	55.66	17.5	93.7	0.0037
APRI	0.839	(0.761, 0.900)	1.5	57.14	95.24	61.5	94.3	<0.0001
FIB-4	0.829	(0.749, 0.891)	3.25	57.14	91.43	47.1	94.1	<0.0001

GP73: Golgi protein 73; APRI: AST-to-platelet ratio index; FIB-4: fibrosis index based on four factors; AUC: area under the curve; CI: confidence interval; PPV: positive predictive value; NPV: negative predictive value.

## Data Availability

The data used to support the findings of this study are available from the corresponding authors upon request.

## Abbreviations

HCV: Hepatitis C virus
CHC: Chronic hepatitis C
APRI: AST-to-platelet ratio index
FIB-4: Fibrosis index based on four factors
TE: Transient elastography
GP73: Golgi protein 73
ALT: Alanine aminotransferase
AST: Aspartate aminotransferase
CLC: Compensated liver cirrhosis
DLC: Decompensated liver cirrhosis
ELISA: Enzyme-linked immunosorbent assay
PEG-IFN: PEGylated interferons
AUROC: Area under the receiver operating characteristic
AUC: Area under the curve
CI: Confidence interval
PPV: Positive predictive value
NPV: Negative predictive value
BMI: Body mass index
TBIL: Total bilirubin
GGT: Gamma glutamyltransferase
ALB: Albumin
GLB: Globulin
RBC: Red blood cell
PLT: Platelet
PT: Prothrombin time
PA: Prealbumin
TBA: Total bile acid
APOE: Apolipoprotein E.
